# ANCA-Associated Vasculitis With Anti-GBM Disease and Two Types of Tumors: A Case Report

**DOI:** 10.3389/fmed.2021.810680

**Published:** 2022-01-21

**Authors:** Xiuxiu Li, Meichun Huang, Jun Liu

**Affiliations:** Tongde Hospital of Zhejiang Province, Hangzhou, China

**Keywords:** glomerular basement membrane (GBM), anti-neutrophil cytoplasmic antibody (ANCA), dual antibodies, colon cancer, lung cancer

## Abstract

**Introduction:**

Anti-neutrophil cytoplasm antibody (ANCA)-associated-vasculitis and anti-glomerular basement membrane (GBM) disease are types of autoimmune diseases that are characterized by the presence of circulating autoantibodies. Most patients with these diseases experience sudden onset, rapid progress, and poor prognosis. The purpose of the present article is to report a case of ANCA-associated vasculitis with anti-GBM disease and two types of tumors.

**Case Report:**

A 63-year-old Chinese woman who underwent resection for rectal cancer 6 years before and for lung adenocarcinoma 4 years before, presented with fever and nasal obstruction, for the past 2 months and chondritis of an ear for the past 1 month. The patient failed to respond to an anti-infection treatment at local and higher-level hospitals with the first episode of “recurrent sinusitis and fever.” Later, systemic symptoms such as fatigue, numbness of the limbs, and auricular chondritis gradually aggravated, followed by an increase in inconspicuous hematuria, proteinuria, and serum creatinine level. After admission, the GBM antibody, C-ANCA, and PR3 were positive. The renal puncture was diagnosed as anti-glomerular basement membrane antibody disease. After treatment, her serum creatinine decreased to 104 umol/l.

**Discussion:**

In the present report, we introduced the case of a rare double-positive disease in a patient with two types of tumors. Importantly, we noted that colon cancer and lung cancer, PR3, and anti-GBM disease may be related to their pathogenesis and manifestations. Further research is warranted to confirm these hypotheses.

## Introduction

Anti-neutrophil cytoplasm antibody (ANCA)-associated-vasculitis and anti-glomerular basement membrane (GBM) disease are types of autoimmune diseases that are characterized by the presence of circulating autoantibodies. Most patients with these diseases experience sudden onset, rapid progress, and poor prognosis. ANCA- associated vasculitis (AAV) with anti-GBM disease is thought to be relatively rare. Patients with AAV exhibit higher rates of malignancy than the general population. Here, we have presented a case of AAV combined with anti-GBM diseases and two types of cancers along with a review of the literature on this important but rare condition.

## Case Report

A 63-year-old Chinese woman who underwent resection for rectal cancer 6 years before and for lung adenocarcinoma 4 years before, presented with fever and nasal obstruction, for the past 2 months and chondritis of an ear for the past 1 month. Chronic sinusitis was diagnosed based on the presenting symptoms and the findings of paranasal sinus radiography and nasal endoscopy. She was accordingly placed on a 2-week course of antibiotics (clarithromycin); however, the symptomes did not recover. The blood test results were as follows: white blood cells 3.63 × 10^9^/L, neutrophil percent 69.9%, and CRP 116.6 mg/L, and her anti-neutrophil cytoplasmic antibody profile indicated cANCA and PR3 positivity, indicating AAV. The rheumatologist reported the following: routine blood tests: hemoglobin 75 g/L; urinalysis revealed urine protein + and negative hematuria; ESR 123 mm/h, ferritin 472.15 ng/mL, albumin 33.1 g/L, serum creatinine 90.1 μmol/L, CRP 111.05 mg/L; and 24-h urine protein quantitative 209.59 mg/24 h. The bone density suggested osteoporosis. No obvious abnormalities were noted in the bone marrow puncture, or serum and urinary protein electrophoresis. AAV was diagnosed, she was intravenously administered methylprednisolone injection (40 mg/day). The patient had repeated episodes of body fever, nasal congestion, and runny nose, accompanied by right earache, dizziness, upper abdominal discomfort, nausea, and vomiting. The patient was hence asked to transfer to our hospital.

The patient had previously undergone rectal cancer resection on September 10, 2015, for which she had received radiotherapy and chemotherapy before the operation. In April 2017, a nodule was detected in her lungs and she accordingly underwent upper-left lung resection. The pathology revealed adenocarcinoma, and no radiotherapy or chemotherapy treatment was performed after the surgery.

Her laboratory studies revealed a serum creatinine (Cr) level of 174 μmol/L (normal range: 40–83 μmol/L), BUN 11.3 mmol/L (normal range: 3.1–8.8 mmol/L), hemoglobin 75 g/L (normal range: 115–150 g/L), CRP 141.2 mg/L, and ESR 144 mm/h. Her urine analysis indicated proteinuria of >840.68 mg/24 h, urine protein (+), red blood cells (++), and no white blood cells in the urine. Her serum albumin level was 40 g/L (normal range: 40–55 g/L). In addition, her ANA was 1:100, PR3 was positive, c-ANCA was 1:100, and serum anti-GBM was positive. P-ANCA, ds-DNA, hepatitis panel, virus antibody, and HIV antibody tests were negative. A renal ultrasound suggested that the kidneys were of normal size. The radiographs of her head, chest, and abdomen, as well as gastroscope and colonoscopy did not suggest any tumor recurrence. After the ophthalmologist's consultation, the patient's eyes were diagnosed with conjunctivitis. Her hearing examination suggested moderate sensorineural hearing loss. A renal biopsy was performed for this patient and the main laboratory results are shown in Table 1.

**Table 1:** Laboratory characteristics at time of kidney biopsy.

**Component**	**Value**	**Reference range**	**Interpretation**
**Urinalysis**			
Urinary protein	+1	Negative	High
Red blood Cells (n/HP)	++	0–3	High
White blood Cells (n/HP)	1–3	0–5	Normal
Urinary protein excretion (g/24 h)	840.68 mg	<200 mg	High
Sugar sediment (mmol/L)	Negative	Negative	Normal
**Hematology**			
White blood cells (×10^9^ /L)	4.3	3.5–9.5	Normal
Neutrophils (×10^9^/L)	3.5	1.8–6.3	Normal
Red blood cells (×10^12^/L)	2.56	3.80–5.10	Low
Hemoglobin (g/L)	75	115–150	Low
Platelets (×10^9^/L)	323	125–350	Normal
**Blood chemistry**			
Serum creatinine (μmol/L)	174	40–83	High
Blood urea nitrogen (mmol/L)	11.3	3.1–8.8	High
Uric acid (μmol/L)	272	140–340	Normal
eGFR (ml/min/1.73 m^2^)	27		low
Serum albumin (g/L)	40	40–55	Normal
Aspartate aminotransferase (U/L)	17	15-35	Normal
Alanine aminotransferase (U/L)	8	7–40	Normal
Lactate dehydrogenase (U/L)	196	120–250	Normal
Alkaline phosphatase (U/L)	85	30–120	Normal
C-reactive protein (mg/L)	141	0–8	High
**Immunology**			
MPO-ANCA	Negative	Negative	Normal
PR3-ANCA	Positive	Negative	High
P-ANCA	Negative	Negative	Normal
C-ANCA	1:100	Negative	High
Anti-GBM antibody	Positive	Negative	High

*eGFR, estimate glomerlular filtration rate; MPO-ANCA, myeloperoxidase-anti-neutrophil cytoplasmic antibodies; PR3-ANCA, proteinase-3-anti-neutrophil cytoplasmic antibodies; p-ANCA, Anti-myeloperoxidase ANCA; c-ANCA, Anti-proteinase-3 ANCA; anti-GBM, anti-glomerular basement membrane*.

## Renal Biopsy

**Figure 1:**
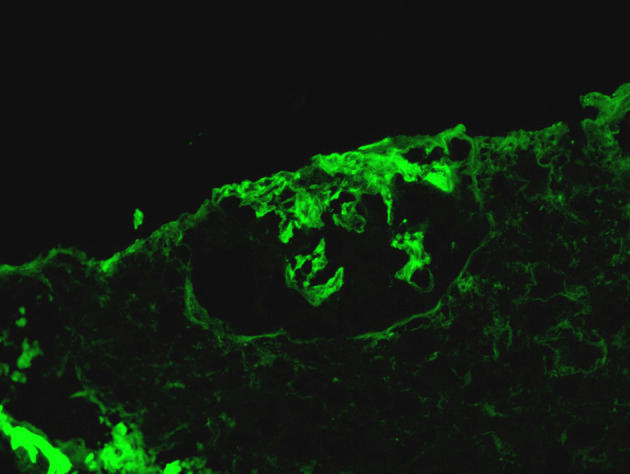
Linear (2+) staining along the glomerular capillary loops for IgG.

Immunofluorescence microscopy demonstrated linear (2+) staining along the glomerular capillary loops for IgG (Figure 1) supported the diagnosis of anti-GBM disease. Half of 5 glomeruli also showed 2+ patchy staining for C3. In addition, 1-2 + patchy mesangial staining for IgM was also noted. There was no evidence of IgA, Kappa, lambda, and C4 or C1q deposition.

**Figure 2:**
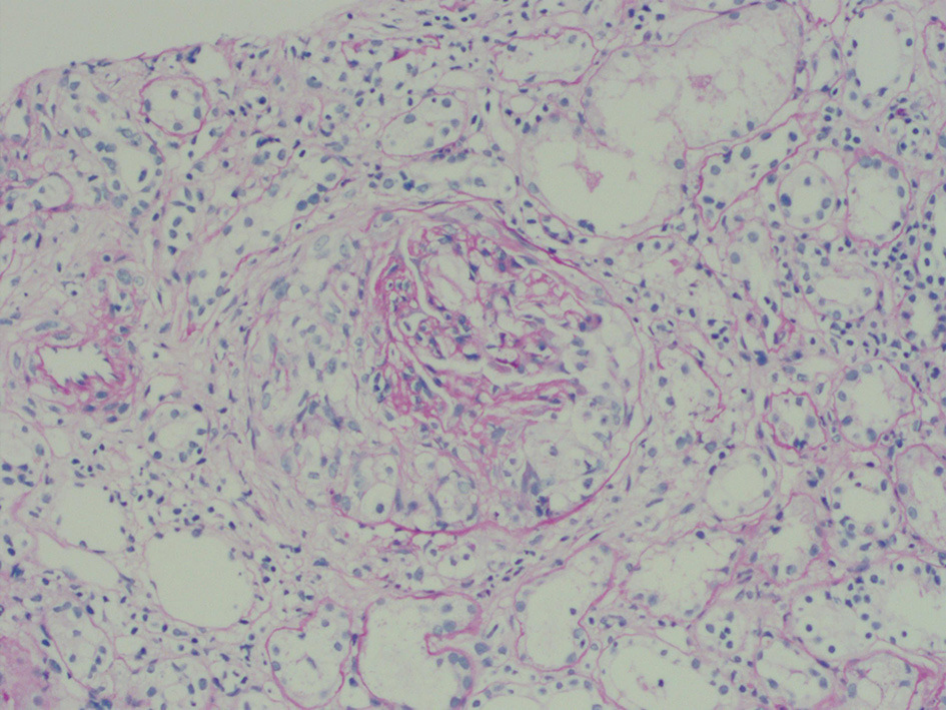
Light microscopy revealed 44 cellular crescents in 72 glomeruli.

Light microscopy revealed 72 glomeruli, of which 8 were globally sclerosis, 2 were segmental sclerosis, 39 were large cellular crescents, 5 were small cellular crescents, cellular crescents account for 61%, which suggested crescentic glomerulonephritis (Figure 2). In addition, the biopsy revealed moderately increased cellularity with both mesangial and segmental endocapillary proliferation. The tubulointerstitial region demonstrated swelling of the tubular epithelial cells, multifocal tubular atrophy (~50%) accompanied by interstitial fibrosis (~15%), moderate interstitial inflammation, and no evidence of extraglomerular vasculitis.

Electron microscopy revealed that the foot processes were diffusely fused, the basement membrane was slightly wrinkled and thickened, the mesangial matrix was increased, and no electron-dense deposits were noted.

**Figure 3:**
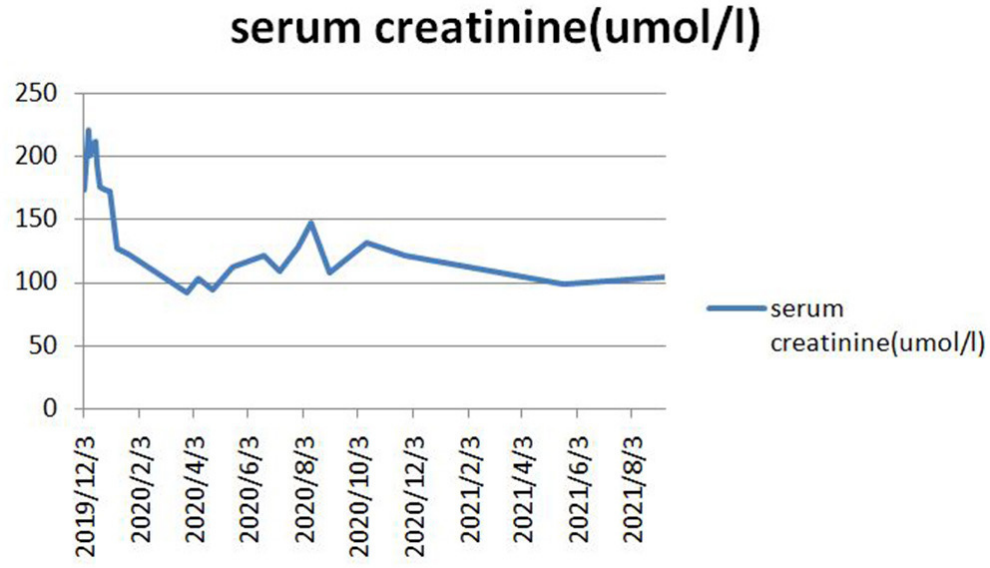
The decline in serum creatinine during treatment.

This patient was immediately administered 250 mg/day methylprednisolone for 2 days and then 60 mg/day intravenously and plasma exchange was given. Plasma exchange was performed 5 times every other day, and a total of 8,020 mL of the plasma was exchanged. Her anti-GBM antibody turned negative, and the blood creatinine level was 176 μmol/L. She was discharged from the hospital with 40 mg/day prednisone and cyclophosphamide orally. The level of steroids gradually decreased, and the cumulative dose of cyclophosphamide was 6.8 g. Because of the decline in the leukocyte and platelet level, we replaced the dosage of the immunosuppressant with mycophenolate mofetil to 0.5 g daily a day 1 year before. Presently, 2.5 mg/day oral prednisone and 0.5 g/day mycophenolate mofetil are being provided. After 2 years of follow-up, her serum creatinine level has slowly decreased to 104 μmol/L, anti-GBM is negative, c-ANCA is 1:10, PR3 is negative, 24-h urine protein level is 209.59 mg/24 h and Hb is 109 g/L. The changes in the serum creatinine level during the treatment are depicted in Figure 3.

## Discussion

Anti-GBM disease has an unknown etiology. It is a rare autoimmune disease that is characterized by the presence of circulating anti-GBM antibodies that interact with the alpha-3 chain of type 4 collagen present in the patient's kidneys and alveoli (1). The prevalence of anti-GBM diseases is ~1/2 million, and the age distribution is bimodal, with the two peak age groups of the disease being ~30 years or 60 years (2).

AAV with the anti-GBM disease is a rare, potentially life-threatening disease. Approximately one-third of all patients with anti-GBM disease have ANCA antibodies, usually P-ANCAs (3). Patients with dual positivity were more likely to have a P-ANCA pattern and a higher specificity for MPO than PR3 (4). However, this patient was c-ANCA and PR3 positive. Then, what is the difference between PR3 and MPO? PR3-ANCA has a younger age of onset, has more acute clinical manifestations, greater respiratory and upper respiratory tract lesions, a greater possibility of relapse, and a better response to rituximab treatment than cyclophosphamide (5).

Generally, both antibodies are found when clinical symptoms appear in patients with rapidly progressing crescentic glomerulonephritis. The current research reports that dual-antibody positive patients experienced varying responses to therapy. O'Donaghue reported 3 cases of double-antibody-positive patients, even if they were treated, all of them eventually depended on dialysis (6). In contrast, the Boschetal indicated that patients with double-antibody positive have better therapeutic effects than patients with only anti-GBM antibodies (7). Another study reported that patients with dual antibody positivity had a better outcome than those with isolated anti-GBM antibodies (8).

Whether ANCAs are non-pathogenic or involved in the complex interaction of neutrophils, macrophages, lymphocytes, cytokines, and endothelial cells remains controversial. These interactions can lead to kidney necrosis and crescent formation and affect the lungs. However, anti-GBM antibodies appear to be pathogenic. Thus, the anti-GBM disease is a rare type of small vessel vasculitis that affects glomerular capillaries (possibly causing glomerular necrosis and crescent formation), pulmonary capillaries (possibly causing alveolar hemorrhage), or both (9). Hence, there seems to be some relationship between ANCAs and the goodpasture antigen (10). Furthermore, past studies have shown that both anti-GBM diseases develop into AAV and that some anti-GBM diseases occur after the onset of AAV (11).

When AKI occurred during admission, the coexistence of ANCA and anti-GBM antibodies was recognized in the patient. Based on her renal biopsy, we diagnosed AKI due to anti-GBM disease rather than due to ANCA-related vasculitis. Her renal dysfunction improved with the administration of plasma exchange and corticosteroid and cyclophosphamide treatment.

The patient reported a history of two types of tumors, but we examined her gastrointestinal endoscopy, bone marrow examination, CT of the head, chest, and abdomen, and B-ultrasound. No obvious evidence of tumor recurrence was noted. This raises the question of whether there is any relationship between tumors and these two diseases.

In some cases of AAV, malignant tumors may play a key role in triggering vasculitis. A recent meta-analysis showed that AAV is associated with an increased risk of cancer, with a standardized incidence ratio of 1.74 (12). Currently, the association between malignancies and anti-GBM disease remains unknown.

Several mechanisms have been reported to explain the association between vasculitis and cancer. Tumor cells may produce an immune response to the vascular endothelium, release various cytokines and cause endothelial damage, induce delayed hypersensitivity through the deposition of tumor proteins on the vascular wall, produce a critical level of circulating immune complexes, and damage capillaries and endothelial cells of the veins (13).

In addition, the association between tumors and anti-GBM disease has never been reported. Cancer has occasionally been associated with paraneoplastic syndromes. In the present case, we cannot deny the possibility that these diseases may have happened coincidentally and that cancer may have triggered an occurrence of ANCAs followed by anti-GBM disease via some pathogenic mechanisms.

The treatment for the dual-positive disease is usually the same as that for isolated anti-GBM nephritis. The conventional treatment methods for the anti-GBM disease include plasma exchange to quickly remove pathogenic autoantibodies and the application of cyclophosphamide and corticosteroids to inhibit any further autoantibody production. High-dose steroids (1 mg/kg), oral cyclophosphamide (2 mg/kg) daily or on alternate days, and therapeutic plasma exchange (TPE) were administered to remove the circulating anti-GBM antibodies until the levels became undetectable for 2–4 weeks. Immunosuppressive treatment typically lasts for 3–6 months. Rituximab has been used successfully in refractory disease treatment (14).

Regardless of receiving plasma exchange or immunosuppressive therapy, patients with serum creatinine values >500 mmol/L are eventually unable to discontinue dialysis. Patients with creatinine values <500 mmol/L show a good prognosis (3). Levy et al. compared double-positive patients with only anti-GBM antibody-positive patients (15). The former were older (median age: 66 vs. 40 years) and required immediate dialysis (70 vs. 55%), and a similar proportion of the patient population had pulmonary hemorrhage (41 vs. 58%) with a similar increase in the mortality rate. Patients with both antibody positives generally have significant systemic inflammation (similar to patients with vasculitis), albeit there are only a relatively few patients with systemic manifestations of vasculitis (such as scleritis, rash, and neuropathy) (3). Here, our patient's clinical symptoms were consistent with AAV, although the renal damage was consistent with that of anti-GBM diseases.

## Conclusion

In the present report, we introduced the case of a rare double-positive disease in a patient with two types of tumors. Importantly, we noted that colon cancer and lung cancer, PR3, and anti-GBM disease may be related to their pathogenesis and manifestations. Further research is warranted to confirm these hypotheses.

## Data Availability Statement

The original contributions presented in the study are included in the article/supplementary materials, further inquiries can be directed to the corresponding author/s.

## Ethics Statement

The studies involving human participants were reviewed and approved by Tongde Hospital of Zhejiang Province Ethics Committee. The patients/participants provided their written informed consent to participate in this study. Written informed consent was obtained from the individual(s) for the publication of any potentially identifiable images or data included in this article.

## Author Contributions

XL was responsible for treating patients and writing articles. MH was responsible for formulating patient's treatment plan and revising articles. JL was responsible for diagnosing kidney pathology. All authors contributed to the article and approved the submitted version.

## Conflict of Interest

The authors declare that the research was conducted in the absence of any commercial or financial relationships that could be construed as a potential conflict of interest.

## Publisher's Note

All claims expressed in this article are solely those of the authors and do not necessarily represent those of their affiliated organizations, or those of the publisher, the editors and the reviewers. Any product that may be evaluated in this article, or claim that may be made by its manufacturer, is not guaranteed or endorsed by the publisher.
